# HCC risk stratification scores: insights from large multi-center national cohort study

**DOI:** 10.1038/s41598-025-34535-w

**Published:** 2026-02-03

**Authors:** Imam Waked, Gamal Esmat, Mohamed Abdallah, Aisha Elsharkawy, Wafaa Elakel, Islam Ammar, Ehab Kamal, Mohamed Hassany, Nabiel Mikhail, Riham Soliman, Wahid Doss, Gamal Shiha

**Affiliations:** 1 National Liver Institute, Menoufia University, Shebeen El Kom, Egypt; 2 National Committee for Control of Viral Hepatitis, MOH, Cairo, Egypt; 3 Endemic Medicine Department, Faculty of Medicine, Cairo University, Cairo, Egypt; 4 Medical Research Division, National Research Center, Giza, Egypt; 5 Hepatogastroenterology and Infectious Diseases, Faculty of Medicine, Al- Zhar University, Cairo, Egypt; 6 National Hepatology and Tropical Medicine Research Institute, Cairo, Egypt; 7 Biostatistics and Cancer Epidemiology Department, South Egypt Cancer Institute, Assiut University, Assiut, Egypt; 8 Egyptian Liver Research Institute and Hospital (ELRIAH), Mansoura, Egypt; 9 Tropical Medicine Department, Faculty of Medicine, Port Said University, Port Said, Egypt; 10 Hepatology and Gastroenterology Unit, Internal Medicine Department, Faculty of Medicine, Mansoura University, Mansoura, Egypt

**Keywords:** Hepatitis C virus, Hepatocellular carcinoma (HCC), Risk scores, SVR, HCC surveillance, Diseases, Gastroenterology, Medical research

## Abstract

Current guidelines advocate biannual HCC surveillance for chronic hepatitis C patients who achieved sustained virological response (SVR) following direct acting antivirals (DAAs), posing a heavy burden on healthcare systems. This study aimed to identify the most accurate and clinically useful risk stratification tool among patients who achieved SVR following DAAs in a large, multicenter national cohort. A retrospective study across 52 NCCVH centers in Egypt included 8,419 CHC patients with cirrhosis (F4) or advanced fibrosis (F3) who achieved SVR after DAAs and had complete follow-up data. Baseline data were used to calculate HCC risk scores (GES, aMAP, THRI, ALBI, FIB-4). Prognostic performance was assessed using Kaplan–Meier analysis, AUC, Harrell’s C-index, and Brier score with calibration plots. Decision curve analysis and Net Reclassification Improvement were applied to evaluate clinical utility and incremental prognostic value. All evaluated HCC risk scores showed adequate performance, with significant separation of incidence curves (log-rank *p* ≤ 0.05). GES demonstrated the highest AUC (0.632), followed by THRI (0.600) and aMAP (0.591), with C-indices around 0.63.Kaplan–Meier analysis confirmed significant survival differences between risk groups. GES had superior calibration (Brier score 0.197, slope 0.90). Decision Curve Analysis favored GES, offering the greatest net clinical benefit, especially at thresholds 0.1–0.3. NRI analysis showed aMAP worsened classification (NRI = − 0.16), while THRI offered minimal improvement over GES (NRI = + 0.05). Implementation of the GES score in Egypt’s national HCC surveillance program, enabling individualized surveillance and reducing healthcare burden in accordance with current EASL recommendations.

**Supplementary Information:**

The online version contains supplementary material available at 10.1038/s41598-025-34535-w.

## Introduction

 Hepatitis C virus (HCV) is a major public health problem with an estimated 50 million people infected worldwide^1^. Treatment with direct acting antivirals (DAAs) have revolutionized the treatment of chronic HCV achieving over 95% efficacy in eradicating all HCV genotypes^2,3^. DAAs reduce, but do not eliminate, the risk of hepatocellular carcinoma (HCC) in those with underlying cirrhosis^4–6^. Current guidelines recommend biannual surveillance in patients who had advanced liver fibrosis and cirrhosis including serial ultrasound imaging ± alpha fetoprotein (AFP) measurements^7,8^. Such an approach encourages earlier identification of dysplastic lesions, broader eligibility for curative therapies and overall improved survival^9^ ; however, a ‘one-size-fits-all’ strategy places a huge burden on healthcare systems. Given the number of HCV-infected individuals who have achieved a sustained virological response (SVR), a more personalized and cost-effective approach to screening is urgently required.

A subsequent policy statement on liver cancer screening from European association for the Study of the Liver (EASL) endorses a risk-based approach and suggests that, approximately 20% of patients who are at low risk could potentially be exempted from regular surveillance. Additionally, it recommends that about 5%–10% of patients at high risk should receive more intensive surveillance, using magnetic resonance imaging (MRI) as the primary surveillance method. Those with an intermediate risk could adhere to the existing guidelines^10^. This strategy has emerged as a more advantageous and not only a cost-effective strategy but also may impose substantial physical harms on patients including multiple CT/MRI. Despite this endorsement, the absence of a defined risk score for patient selection remains a gap in the literature.

Egypt launched a national hepatitis C virus treatment program, through which about 5 million patients received treatment. Approximately 20% of these treated individuals had liver cirrhosis (F4), and another 16% presented with advanced liver fibrosis (F3) resulting in a substantial population requiring HCC surveillance^11^. This increased surveillance demand significantly burdens the healthcare system. Therefore, individualizing HCC risk assessment through established risk prediction scores could effectively reduce this burden. Several validated HCC risk prediction scores, including ALBI^12^, aMAP^13^, GES^14^, and THRI^15^, have been developed using readily available clinical variables. These scores are practical for routine clinical application as they do not rely on expensive molecular or genetic markers or complex computational methods. The objective of our study is to evaluate the predictive performance and clinical utility of these HCC risk prediction scores among patients with cured HCV infection and compensated advanced chronic liver disease (cACLD) in a large, multicenter national cohort, aiming to identify the most accurate and clinically useful risk stratification tool.

## Patients and methods

### Cohort

Out of 33,797 CHC patients, with liver cirrhosis (F4) or advanced liver fibrosis (F3) who had a sustained virologic response (SVR) after receiving DAAs, 8419 patients who completed follow up and had complete data available for scoring parameters were included in this observational study. Between January 2016 and December 2022, patients were recruited from the 52 centers belonging to the National Committee for Control of Viral Hepatitis (NCCVH) throughout Egypt. This study was conducted in accordance with the protocol and the principles of the Declaration of Helsinki [CIOMS/WHO, 1993] and its amendments in 2008^16^. The protocol was approved by the ethical Committee of the National Committee for the Control of Viral Hepatitis (NCCVH), Ministry of Health and Population, Egypt. The need to obtain informed consent from the participants was waived by the IRB due to the retrospective nature of the study.

Patients with decompensated liver disease, evidence of current HCC or focal hepatic lesions, history of HCC or other malignancies and chronic debilitating diseases as chronic kidney diseases or chronic cardiac failure were excluded from the study. All patients in the cohort had their base line data recorded, together with the data in the follow-up visits, up to the last follow-up. HCC incidence was also recorded. For score comparison, we depended on pre-treatment data (immediately before the onset of DAAs).

### Diagnosis of fibrosis and HCC

Patients were diagnosed with advanced liver fibrosis (F3) or cirrhosis (F4) based on a FIB-4 in addition to clinical as follows:

- F3 (advanced fibrosis): defined by a FIB-4 score > 3.25 in the absence of clinical or radiological evidence of cirrhosis.
- F4 (cirrhosis): defined by the same FIB-4 cut-off (> 3.25) *plus* at least one of the following: platelet count < 150 × 10⁹/L, splenomegaly, or evidence of portal hypertension/varices on imaging or endoscopy or evidence of liver cirrhosis on imaging.

Multiphase CT or MRI was performed in patients with focal hepatic lesions detected on abdominal ultrasound and/or an AFP level > 20 ng/mL, to assess for hepatocellular carcinoma based on characteristic arterial phase enhancement and washout in the delayed phase, in accordance with EASL guidelines^17^ .

### Calculation of HCC risk scores

We employed conventional HCC prediction scores including THRI, aMAP, ALBI, GES, and FIB-4. The calculation methods and criteria for stratifying patients into low-, intermediate-, or high-risk categories were adopted from the original peer-reviewed publications that introduced and validated these scores; GES score^14^, FIB-4^18^, ALBI^13^, aMAP score^13^, THRI score^15^. Table 1 &Supplementary material (1).

**Table 1:** Baseline characteristics of the cohort.

Variable	NCCVH Cohort
Patient number	8558
Age (years)	60.0 (53.0–67.0)
Sex	
Males	3724 (43.5)
Females	4834 (56.5)
ALT (U/L)	42.0 (23.0–65.0)
AST (U/L)	45.0 (30.0–65.0)
Total Bilirubin (mg/dL)	0.90 (0.70–1.30)
Albumin (g/dL)	3.9 (3.5–4.2)
Platelets count (/cmm^3^)	150.0 (122.0–185.0)
AFP (ng/ml)	3.48 (2.30–5.40)
Comorbidities	
Yes	576 (6.7%)
No	7982 (93.3%)
FIB4	2.84 (1.81–4.02)
Follow up duration (months, mean ± SD)	25.79 ± 12.90

### Statistical analysis

To evaluate the prognostic performance of the three HCC risk scores—GES, aMAP, and THRI—in predicting hepatocellular carcinoma (HCC), we conducted a multi-step statistical analysis. Follow-up duration was defined as the time from the end of treatment to the date of last follow-up or HCC occurrence, whichever came first. Time-to-event and cumulative incidence analyses were performed using the Kaplan–Meier method. Incidence curves were then compared across the different risk scores using the log-rank (Mantel–Cox) test. Discrimination was evaluated using the area under the receiver operating characteristic curve (AUC) and Harrell’s C-index, both of which quantify the ability of each score to distinguish between patients who developed HCC and those who did not^19,20^. Calibration was assessed using Brier score, calibration plots, calibration slope and the Hosmer-Lemshow to evaluate the accuracy of predicted probabilities^21^. Decision curve analysis (DCA) was performed to examine the net clinical benefit across a range of threshold probabilities, comparing each score to “treat all” and “treat none” strategies^22^. Negative Predictive Value (NPV) was calculated as the proportion of patients classified as low-risk who did not develop HCC during follow-up (true negatives / [true negatives + false negatives]), to evaluate each score’s ability to reliably exclude future HCC occurrence.Net Reclassification Improvement (NRI) was calculated to assess the added value of THRI and aMAP over GES using clinically relevant risk categories^23^. Analyses were conducted using SPSS v26, R v4.3.2, and Python v3.11.A p-value < 0.05 was considered statistically significant.

## Results

Analysis included 8419 patients. Characteristics of studied patients are presented in Table 2. 3672 (43.6%) patients were males while 4747 (56.4%) were females. Median age was 60.0 years (53.0–67.0). Patients were followed up for a mean duration of 25.9 ± 12.9 months (range 12–92 months). 52 patients developed HCC during follow up. Incidence of HCC was 0.29 / 100 py (95% CI = 0.22–0.37). Performance of HCC prediction scores is presented in Tables 2 and 3; Fig. 1 and Supplementary Fig. 1.

**Table 2:** Performance of different risk scores.

Score	Risk group	No. / group (%)	HCC / group	HCC IR / 100 py (95% CI)
GES score	Low	6358 (74.3%)	33	0.24 (0.17-0.34)
	Intermediate	986 (11.5%)	7	0.32 (0.14-0.64)
	High	1214 (14.2%)	16	0.60 (0.35-0.95)
FIB-4	Low	1334 (15.6%)	14	0.53 (0.29-0.86)
	Intermediate	3602 (42.1%)	11	0.15 (0.08-0.26)
	High	3622 (42.5%)	31	0.38 (0.26-0.53)
ALBI	Low	3666 (43.4%)	24	0.31 (0.21-0.46)
	Intermediate	4373 (51.8%)	43	0.44 (0.32-0.60)
	High	399 (4.7%)	3	0.37 (0.09-0.99)
aMAP	Low	1266 (15.0%)	5	0.19 (0.07-0.42)
	Intermediate	3763 (44.7%)	17	0.21 (0.13-0.33)
	High	3390 (40.3%)	30	0.41 (0.28-0.57)
THRI	Low	2515 (29.4%)	7	0.13 (0.06-0.26)
	Intermediate	5206 (60.8%)	41	0.37 (0.27-0.49)
	High	837 (9.8%)	8	0.44 (0.20-0.83)

**Table 3:** Accuracy for prediction of hepatocellular carcinoma development in CHC patients using different scores.

Score	AUROC (95% CI)	Sensitivity^#^ (95% CI)	Specificity^#^ (95% CI)	PPV^#^ (95% CI)	NPV^#^ (95% CI)	Accuracy^#^ (95% CI)	Incidence comparison by Log Rank *p*	Overall performance by Brier Score#	Discrimination by Harrell’s c	Calibration by Hosmer-Lemeshow test
GES score	0.629 (0.553-0.704) *P*=0.001	41.1 (29.2-54.1)	74.4 (73.5-75.3)	1.0 (0.7-1.6)	99.5 (99.3-99.6)	74.2 (73.2-75.1)	<0.001	0.258	0.683	0.705
FIB-4	0.543 (0.452-0.633) *P*=0.270	55.4 (42.4-67.6)	57.8 (56.7-58.8)	0.9 (0.6-1.2)	99.5 (99.3-99.7)	57.8 (56.7-58.8_	0.003	0.422	0.618	<0.001
ALBI	0.563 (0.481-0.644) *P*=0.118	67.3 (53.8-78.5)	43.5 (42.5-44.6)	0.7 (0.5-1.0)	99.5 (99.3-99.7)	43.7 (42.6-44.7)	0.393	0.563	0.556	1.000
aMAP	0.623 (0.546-0.701) *P*=0.002	90.4 (79.4-95.8)	15.1 (14.3-15.9)	0.7 (0.5-0.9)	99.6 (99.1-99.8)	15.5 (14.8-16.3)	0.041	0.845	0.595	0.427
THRI	0.632 (0.567-0.697) *P*=0.001	87.5 (76.4-93.8)	29.5 (28.5-30.5)	0.8 (0.6-1.07)	99.7 (99.4-99.9)	29.9 (28.9-30.9)	0.019	0.701	0.597	1.000

# comparing risky patients (high + intermediate risk groups) with less risky patients (low risk group).

**Fig. 1:**
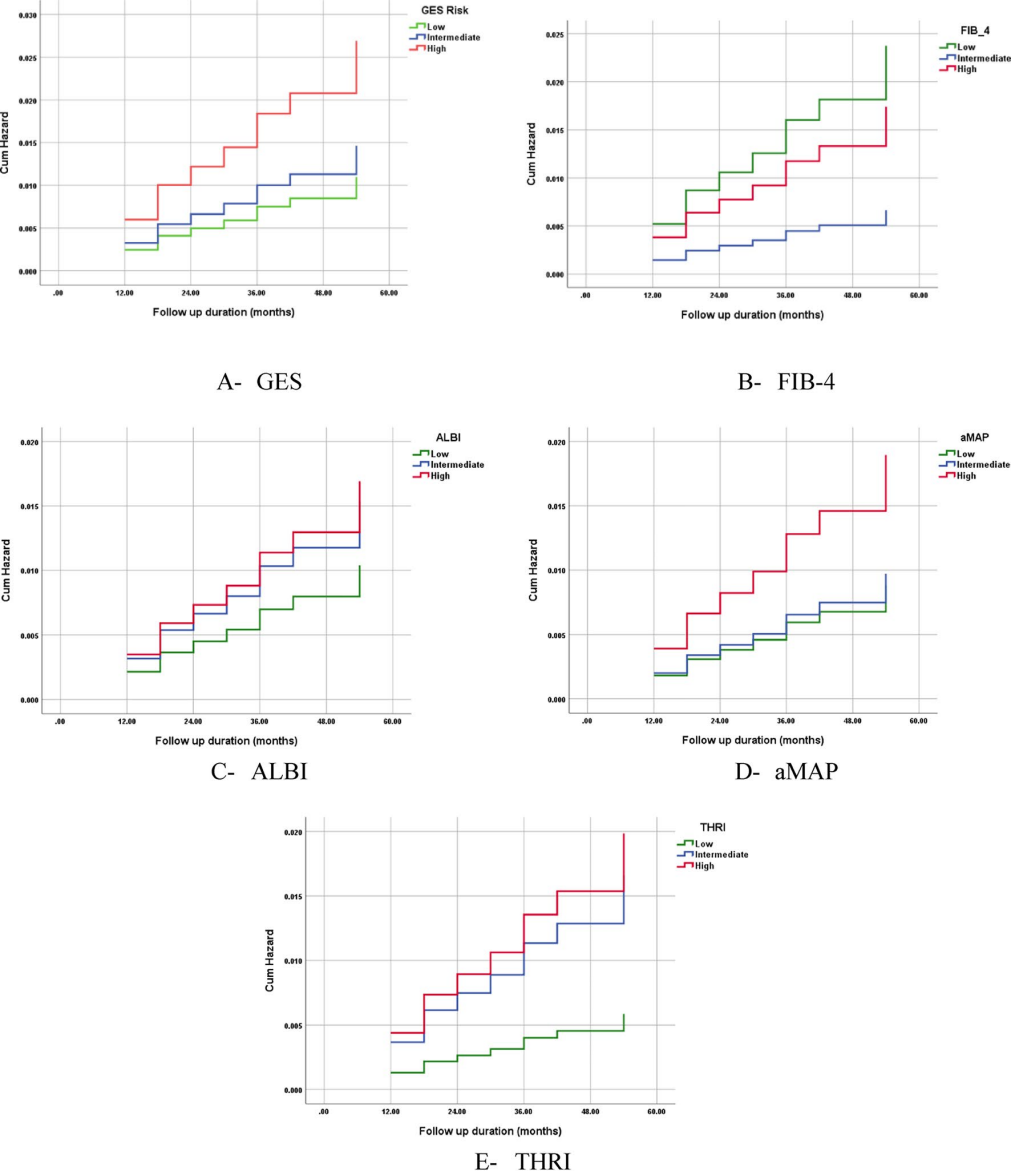
Cumulative incidence of hepatocellular carcinoma according to risk stratification by prediction scores.

### ALBI grade

Patients were classified by ALBI into low-risk group (3658 patients, 43.4%), intermediate risk group (4362 patients, 51.9%) and high-risk group (399 patients, 4.7%). HCC developed in 17 of patients belonging to low-risk group with incidence of 0.22 / 100 py (95% CI = 0.13–0.35), 32 belonging to intermediate risk group with incidence of 0.33 / 100 py (95% CI = 0.23–0.46) and 3 belonging to high-risk group with incidence of 0.37 / 100 py (95% CI = 0.09–0.99). Log rank test for comparison of incidence curves is not statistically significant (*p* = 0.393), and Harrell’s c statistics was low (0.556). NPV to rule out occurrence of HCC is 99.1% (95% CI = 98.7–99.3).

## aMAP score

Patients were classified by aMAP into low-risk group (1266 patients, 15.0%), intermediate risk group (3763 patients, 44.7%) and high risk group (3390 patients,40.3%). HCC developed in 5 of patients belonging to low-risk group with incidence of 0.19 / 100 py (95% CI = 0.07–0.42), 17 belonging to intermediate risk group with incidence of 0.21 / 100 py (95% CI = 0.13–0.33) and 30 belonging to high risk group with incidence of 0.41 / 100 py (95% CI = 0.28–0.57). Log rank test for comparison of incidence curves is statistically significant (*p* = 0.041), and Harrell’s.

c statistics was low (0.595). NPV to rule out occurrence of HCC is 96.4% (95% CI 95.3–97.3).

## FIB-4 index

Patients were classified by FIB-4 into low risk group (1282 patients, 15.2%), intermediate risk group (3549 patients, 42.2%) and high risk group (3588 patients, 42.6%). HCC developed in 13 of patients belonging to low risk group with incidence of 0.50 / 100 py (95% CI = 0.28–30.84), 10 belonging to intermediate risk group with incidence of 0.13 / 100 py (95% CI = 0.13–0.24) and 29 belonging to high risk group with incidence of 0.36 / 100 py (95% CI = 0.24–0.51). Log rank test for comparison of incidence curves is highly statistically significant (*p* = 0.003), and Harrell’s c statistics was fair (0.623). NPV to rule out occurrence of HCC is 99.4% (95% CI = 99.1–99.6).

### GES score

Patients were classified by GES score into low risk group (6234 patients, 74.0%), intermediate risk group (982 patients, 11.7%) and high risk group (1203 patients, 14.3%). HCC developed in 30 of patients belonging to low risk group with incidence of 0.22 / 100 py (95% CI = 0.15–0.32), 6 belonging to intermediate risk group with incidence of 0.28 / 100 py (95% CI = 0.13–0.58) and 16 belonging to high risk group with incidence of 0.60 / 100 py (95% CI = 0.36–0.95). Log rank test for comparison of incidence curves is highly statistically significant (*p* = 0.004), and Harrell’s c statistics was fair (0.681). NPV to rule out occurrence of HCC is 99.7% (95% CI = 99.5–99.8).

### THRI score

Patients were classified by THRI into low-risk group (2453 patients, 29.1%), intermediate risk group (5137 patients, 61.1%) and high-risk group (829 patients, 9.8%). HCC developed in 6 of patients belonging to low-risk group with incidence of 0.11 / 100 py (95% CI = 0.05–0.23), 38 belonging to intermediate risk group with incidence of 0.34 / 100 py (95% CI = 0.25–0.47) and 8 belonging to high risk group with incidence of 0.44 / 100 py (95% CI = 0.20–0.84). Log rank test for comparison of incidence curves is statistically significant (*p* = 0.015), and Harrell’s c statistics was fair (0.605). NPV to rule out occurrence of HCC is 98.2% (95% CI = 97.5–98.6).

### Comparison of HCC prediction scores (Table 3; Fig. 1)

Except ALBI, all HCC risk scores investigated had adequate statistical performance with significant Log rank (Mantel–Cox) analysis for comparison of incidence curves (p value ≤ 0.05).

GES demonstrated the highest area under the receiver operating characteristic curve (AUC = 0.632), followed by THRI (AUC = 0.600) and aMAP (AUC = 0.591). supplementary Fig. 1& figure Kaplan–Meier analysis demonstrated significant separation in HCC-free survival among patients stratified into low-risk versus intermediate/high-risk groups by each score. The log-rank (Mantel–Cox) test confirmed that these differences were statistically significant (*p* < 0.05), supporting the utility of all three scores in stratifying risk over time.

In terms of discrimination, Harrell’s C-index values for time-to-event data were similar across the models, with each score achieving a C-index of approximately 0.63, indicating modest but consistent ability to distinguish between individuals who did and did not develop HCC during follow-up.

**Fig. 2:**
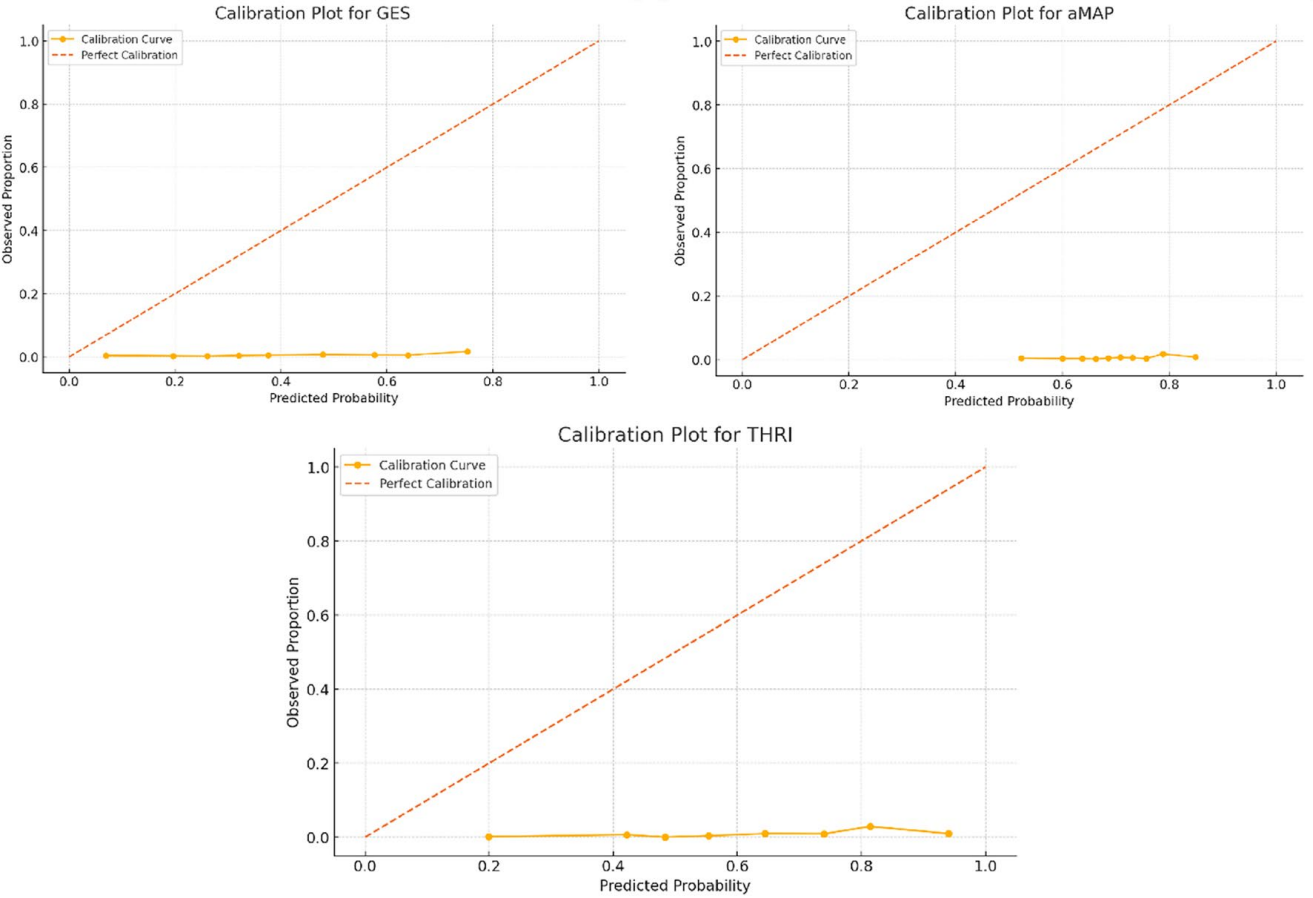
Calibration plots.

In terms of calibration, GES demonstrated the most accurate probability estimates, with the lowest Brier score (0.197), compared to THRI (0.325) and aMAP (0.487). The calibration slope for GES was 0.90 with an intercept of − 5.00, indicating slight overestimation of risk but overall good calibration. In contrast, THRI and aMAP exhibited substantial miscalibration, with slopes of − 0.008 and 0.26, and intercepts of − 6.49 and − 5.32, respectively. Visual inspection of calibration plots confirmed that GES provided predictions closest to the ideal 45-degree line, particularly in the mid-risk range. Although none of the scores achieved perfect calibration, GES showed the least deviation from observed outcomes. Figures 2.

**Fig. 3:**
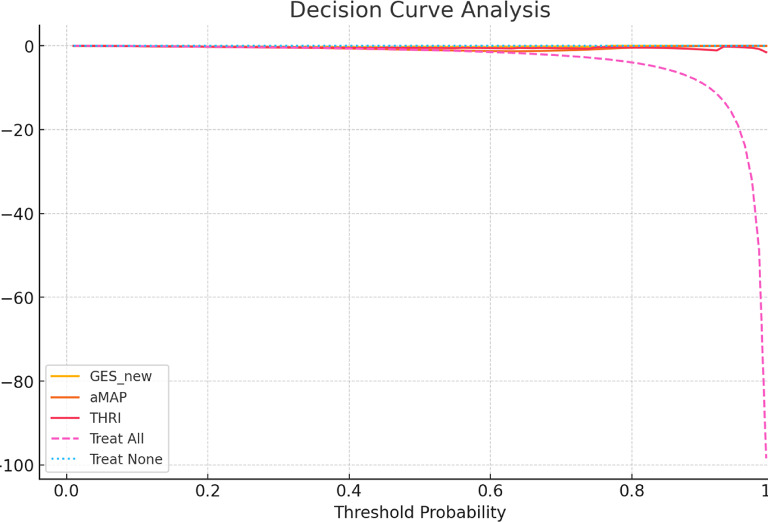
Decision curve analysis.

**Fig. 4:**
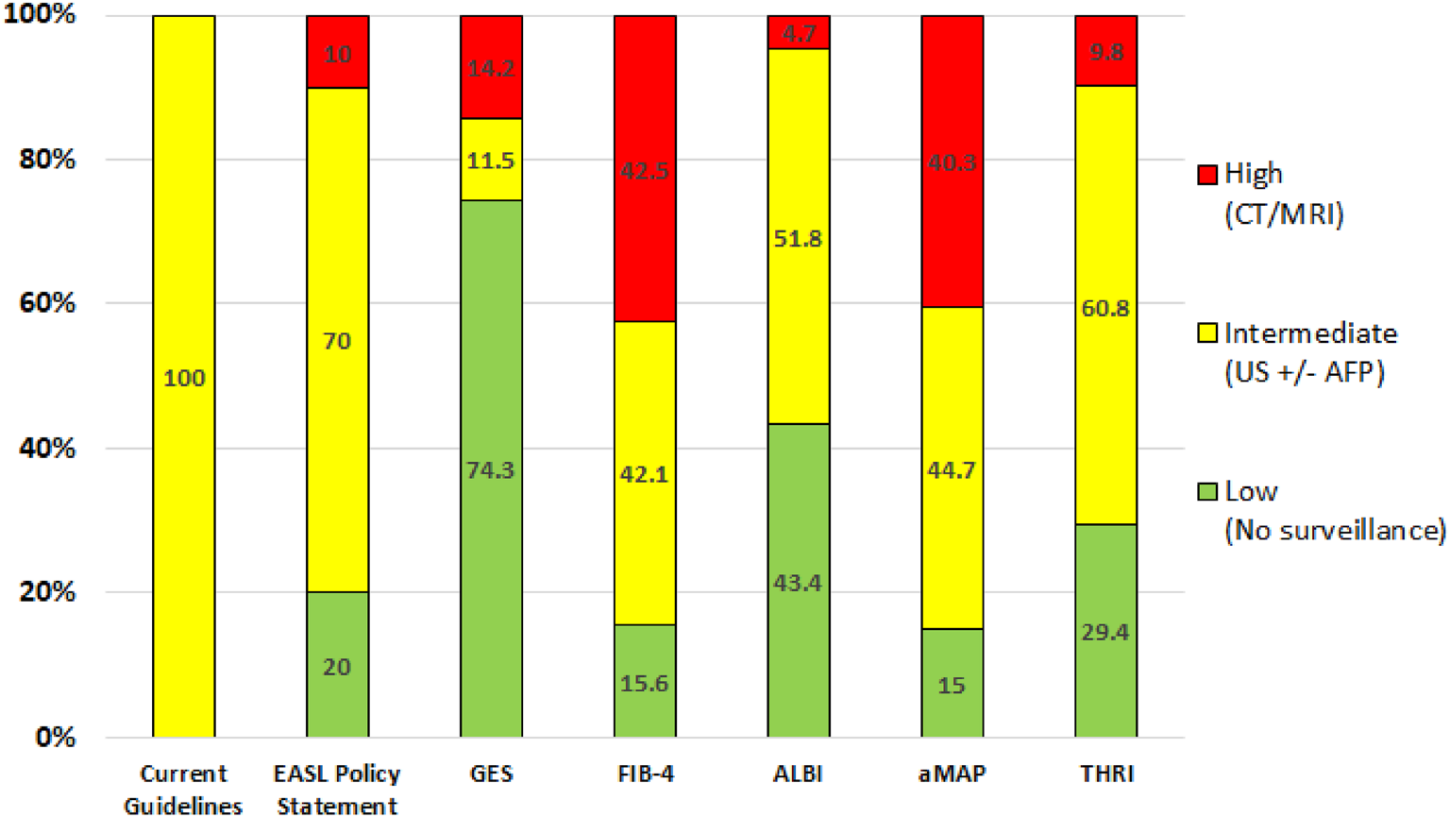
Patient risk distribution by prediction scores compared to EASL policy statement and current guidelines.

Decision Curve Analysis (DCA) demonstrated that the GES score provided the highest net benefit across the clinically relevant threshold range (0.1–0.5), consistently outperforming both the “treat-all” and “treat-none” strategies. In contrast, THRI showed moderate net benefit, while aMAP offered limited utility, particularly at the lower end of the threshold range. Within the lower thresholds (0.1–0.3), where early intervention is most impactful, GES maintained a consistently superior net benefit, supporting its clinical utility in early risk stratification and guiding individualized HCC surveillance decisions. Figure 3and 4.

Net reclassification improvement (NRI) analysis revealed that replacing GES with aMAP resulted in poorer classification performance, with a negative NRI of − 0.16, primarily due to inferior classification of non-events. In contrast, THRI offered only a marginal improvement over GES, with a modestly positive NRI of + 0.05, insufficient to warrant preference over GES in clinical practice.

## Discussion

Our study showed that all three scores; aMAP, GES and THRI were able to stratify our patients into low, intermediate and high-risk groups. Notably, the low-risk group exhibited a remarkably low cumulative incidence rate: 0.22/100 person-years (py) for GES, 0.19/100 py for aMAP, and 0.11/100 py for THRI. Among these, the GES score identified the largest proportion of patients (74.0%) as low risk, substantially higher than aMAP (15.0%) and THRI (29.1%). This finding has important clinical and economic implications, as the application of GES may allow a substantial proportion of patients to undergo extended HCC surveillance intervals, thereby improving the cost-effectiveness of HCC monitoring programs.

It should be noted that aMAP classified a markedly higher proportion of patients (40.3%) as high risk, compared to 14.3% with GES and 9.8% with THRI. While aMAP’s broader high-risk categorization may enhance sensitivity, it could also pose significant challenges in terms of healthcare resource utilization, potentially limiting its feasibility in routine clinical practice.

The aMAP was validated in 2085 genotype-4 HCV-cured patients with advanced liver diseases and exhibited strong statistical performance. However, the authors noted that the aMAP categorized approximately 85% of their cohort into the high-risk group, which could potentially reduce the cost-effectiveness of the surveillance program and pose significant physical risks to the patients^24^. Recently, the aMAP score was compared to the newly developed SMART model by Minami et al. where the SMART model showed superior discriminative performance, with a higher c-index than aMAP in both the derivation cohort (0.936 vs. 0.762) and the validation cohort (0.839 vs. 0.830)^25^.

The Toronto Hepatocellular Carcinoma Risk Index has been validated within a single Asian cohort comprising individuals with cirrhosis of varying etiologies. The authors of this study concluded that the score effectively stratified patients into three distinct risk groups, with only 5.2% of patients falling into the low-risk category^26^. In a Swedish context, Astrom et al., reached the conclusion that the THRI could successfully distinguish between individuals at low and high risk of developing HCC. However, it is worth noting that the low-risk group was relatively small, accounting for just 5.3% of the total cohort, which may limit the clinical utility of the THRI^27^.

When evaluating the individual predictive performance of the GES, aMAP, and THRI scores, notable differences emerged in both discrimination and calibration. The GES demonstrated the highest discriminatory ability (AUC = 0.632), followed by THRI (AUC = 0.600) and aMAP (AUC = 0.591). These values indicate that all three models have modest capacity to distinguish between patients who will develop hepatocellular carcinoma (HCC) and those who will not. However, when calibration was assessed using the Brier score to evaluate the accuracy of predicted probabilities, GES outperformed the other scores. It achieved the lowest Brier score (0.197), reflecting more accurate and reliable risk estimates compared to THRI (0.325) and aMAP (0.487).

It also delivered the highest net clinical benefit across relevant decision thresholds and exhibited greater consistency in reclassification performance. These results support the use of GES as the most clinically reliable and actionable tool for individualized HCC risk estimation in this patient population.

The discriminative capability of the GES score was validated in a multicentre international cohorts, encompassing diverse populations from Europe, Japan, India, and the United States. The authors asserted that the GES score effectively categorizes HCV patients into three risk groups for HCC. Furthermore, it exhibited significant predictive efficacy for HCC development across all participants (*p* < 0.0001), with a Harrell-C index ranging from 0.55 to 0.76 across all cohorts, even after adjustments for HCV genotypes and patient ethnicities^28^. The dynamics of the GES were also externally validated in a Japanese cohort with genotypes 1 and 2. In this study, Abe et al. concluded that the GES demonstrated excellent performance in HCC risk stratification for DAA-treated HCV patients with other genotypes, yielding a fair Harrell-C index^29^. Similar findings were reported by Muzica et al. who compared several HCC risk scores among 992 Romanian CHC patients and concluded that GES score has a very good predictive power for the risk of HCC post- SVR and could be recommended in clinical practice in Romaina^30^. In line with these findings, Qureshi et al., supported the utility of GES as a simple and practical tool for optimizing HCC surveillance strategies in high-risk, post-SVR Pakistani patients with HCV-genotype 3^31^.

Moreover, we recently reported the outcomes of the first prospective study evaluating individualized risk stratification using GES in CHC patients who achieved sustained virologic response (SVR) at Tanta University Hospitals. This prospective cohort study included 492 patients with a mean follow- up duration of 2 years and demonstrated that the implementation of individualized risk stratification using GES significantly enhanced early detection of hepatocellular carcinoma (HCC). Notably, 80% of detected HCC cases were identified at early, potentially curable stages (BCLC stages 0 and A), a substantial improvement compared to only 52% in the comparative cohort following regular surveillance. These findings provide compelling evidence supporting the use of individualized risk stratification with GES to optimize surveillance strategies and increase opportunities for curative treatment^32^.

The superior performance of the GES_new score may be attributed to its incorporation of biologically and clinically relevant variables, such as AFP and fibrosis stage, which directly reflect tumor risk and liver disease progression. In contrast, aMAP and THRI lack AFP and rely on indirect or non-specific markers. Moreover, GES includes albumin but not platelet count, prioritizing hepatic synthetic function over surrogate markers of portal hypertension. Recent studies^33,34^ have demonstrated that monitoring longitudinal changes in AFP, rather than relying on a single absolute cut-off value, can substantially enhance the sensitivity of HCC detection. In line with these findings and our own results, we suggest that an effective HCC risk prediction score should incorporate AFP dynamics during follow-up. This is further supported by the observation that existing scores such as the aMAP and THRI—which do not include AFP—were not clinically useful in our patient cohort. Notably, to address this limitation, Fan et al. recently incorporated AFP into the aMAP model to develop aMAP-2, a novel score that integrates longitudinal AFP and aMAP trends, thereby improving HCC risk prediction and overcoming the static nature of the original aMAP score^35^.

Our study offers several strengths that enhance its validity and clinical relevance. It represents the first large-scale, multicentre national study directly comparing the predictive performance of multiple HCC risk stratification scores—including aMAP, GES, and THRI—in a well-defined cohort of patients with advanced fibrosis or cirrhosis who achieved sustained virologic response (SVR) after DAA therapy. The inclusion of over 8,000 patients and a long follow-up period provides robust statistical power and allows for meaningful incidence estimation of HCC in this population. Additionally, the real-world nature of the cohort, drawn from 52 centres across Egypt, enhances generalizability within similar populations.

Our study also had limitations; First, the retrospective design carries inherent risks of selection and confounding bias, despite the large sample size and systematic data collection. Second, all patients were recruited from the Egyptian National Liver Disease Program, which may limit generalizability to other healthcare settings such as primary care or to populations outside Egypt. The predominance of HCV genotype 4 in our cohort, together with local environmental and lifestyle factors, may influence the risk profile for HCC and differ from Asian or Western populations where other genotypes are more common. Another limitation of this study is that it does not address recent accurate prediction scores based on molecular and genetic risk factors, such as the hepatic fat genetic risk score or the TLL1 variant. However, these scores are costly and not practical for implementation in a national program. Moreover, our study relied on conventional clinical indicators in risk scores, without incorporating emerging biomarkers such as des-γ-carboxy prothrombin (DCP) or glypican-3. Although AFP is included in the GES score, longitudinal AFP dynamics and integration with novel biomarkers may further enhance predictive accuracy. Future studies should evaluate models that combine these markers with clinical variables to optimize HCC risk stratification. A further limitation is that detailed information on non-HCC causes of death was not available in our dataset, which precluded the use of competing risks methods such as the Fine–Gray model. As a result, our Kaplan–Meier estimates may slightly overestimate the cumulative incidence of HCC. However, prior evidence suggests that risk score stratification remains robust when competing risks are considered. Additional limitation is the variability in surveillance practices and suboptimal adherence to recommended intervals—an acknowledged global challenge—may have influenced early HCC detection. The relatively limited follow-up time, some patient losses, and occasional delays or restricted access to definitive imaging could also have affected incidence estimates. These factors should be considered when interpreting our results.

In conclusion, the implementation of the GES score within this national hepatitis C virus (HCV) elimination program effectively stratifies patients according to hepatocellular carcinoma (HCC) risk. Notably, the GES score categorized the largest proportion of patients (74.3%) as low-risk, allowing longer follow-up intervals. Adopting GES in routine clinical practice, may offer more favorable harm-benefit tradeoffs, helping clinicians better identify high-risk individuals for hepatocellular carcinoma surveillance or early intervention while minimizing unnecessary procedures in low-risk patients.

## Supplementary Information

Below is the link to the electronic supplementary material.

Supplementary Material 1

## Data Availability

The data of this study is available from the corresponding author upon request.

## References

[1] Polaris Observatory, H. C. V. & Collaborators Global change in hepatitis C virus prevalence and cascade of care between 2015 and 2020: a modelling study. *Lancet Gastroenterol. Hepatol.* **7** (5), 396–415. 10.1016/S2468-1253(21)00472-6 (2022). 35180382 10.1016/S2468-1253(21)00472-6

[2] Asselah, T., Marcellin, P. & Schinazi, R. F. Treatment of hepatitis C virus infection with direct-acting antiviral agents: 100% cure? *Liver Int.* **38** (Suppl 1(Suppl 1), 7–13 (2018). 29427484 10.1111/liv.13673 PMC7713514

[3] Waked, I. et al. Ombitasvir, paritaprevir, and Ritonavir plus ribavirin for chronic hepatitis C virus genotype 4 infection in Egyptian patients with or without compensated cirrhosis (AGATE-II): a multicentre, phase 3, partly randomised open-label trial. *Lancet Gastroenterol. Hepatol.* **1** (1), 36–44 (2016). 28404110 10.1016/S2468-1253(16)30002-4

[4] EB, G. S. M. Sofosbuvir plus ribavirin for Treatment-Naïve chronic HCV genotype 4 patients: Real-life experience. *Med. J. Viral Hepat.* **2** (1), 1–8 (2016).

[5] Nahon, P. et al. Eradication of hepatitis C virus infection in patients with cirrhosis reduces risk of liver and Non-Liver complications. *Gastroenterology* **152** (1), 142–156e2 (2017). 27641509 10.1053/j.gastro.2016.09.009

[6] Shiha, G. et al. Incidence of HCC in chronic hepatitis C patients with advanced hepatic fibrosis who achieved SVR following daas: A prospective study. *J. Viral Hepat.* **27** (7), 671–679 (2020). 32050037 10.1111/jvh.13276

[7] European Association for the Study of the Liver. EASL recommendations on treatment of hepatitis C 2018. *J. Hepatol.* **69** (2), 461–511 (2018). 29650333 10.1016/j.jhep.2018.03.026

[8] Chung, R. T. et al. Hepatitis C guidance 2018 update: AASLD-IDSA recommendations for Testing, Managing, and treating hepatitis C virus infection. *Clin. Infect. Dis.* **67** (10), 1477–1492 (2018). 30215672 10.1093/cid/ciy585 PMC7190892

[9] Mittal, S. et al. Effectiveness of surveillance for hepatocellular carcinoma in clinical practice: A united States cohort. *J. Hepatol.* **65** (6), 1148–1154 (2016). 27476765 10.1016/j.jhep.2016.07.025 PMC5322857

[10] EASL Policy Statement on liver cancer screening. Risk-based surveillance for hepatocellular carcinoma among patients with cirrhosis. (2023).

[11] Waked, I. et al. Screening and treatment program to eliminate hepatitis C in Egypt. *N Engl. J. Med.* **382** (12), 1166–1174 (2020). 32187475 10.1056/NEJMsr1912628

[12] Fujita, K. et al. Albumin-bilirubin score indicates liver fibrosis staging and prognosis in patients with chronic hepatitis C. *Hepatol. Res.* **49** (7), 731–742 (2019). 30892804 10.1111/hepr.13333 PMC6851801

[13] Fan, R. et al. aMAP risk score predicts hepatocellular carcinoma development in patients with chronic hepatitis. *J. Hepatol.* **73** (6), 1368–1378 (2020). 32707225 10.1016/j.jhep.2020.07.025

[14] Shiha, G. et al. GES: A validated simple score to predict the risk of HCC in patients with HCV-GT4-associated advanced liver fibrosis after oral antivirals. *Liver Int.* **40** (11), 2828–2833 (2020). 32946647 10.1111/liv.14666

[15] Sharma, S. A. et al. Toronto HCC risk index: A validated scoring system to predict 10-year risk of HCC in patients with cirrhosis. *J. Hepatol.* ; (2017). 10.1016/j.jhep.2017.07.033 28844936

[16] International ethical guidelines. *For Biomedical Research Involving Human Subjects* 112 (CIOMS, 2002). 14983848

[17] European Association for the Study of the Liver. EASL clinical practice guidelines: management of hepatocellular carcinoma. *J. Hepatol.* **69** (1), 182–236 (2018). 29628281 10.1016/j.jhep.2018.03.019

[18] Chang, S. H. et al. Fibrosis-4 index stratifies risks of hepatocellular carcinoma in patients with chronic hepatitis C. *J. Formos. Med. Assoc.* **123** (6), 1154–1160 (2024). 38944614 10.1016/j.jfma.2024.06.008

[19] Hanley, J. A. & McNeil, B. J. The meaning and use of the area under a receiver operating characteristic (ROC) curve. *Radiology* **143** (1), 29–36 (1982). 7063747 10.1148/radiology.143.1.7063747

[20] Harrell, F. E., Califf, R. M., Pryor, D. B., Lee, K. L. & Rosati, R. A. Evaluating the yield of medical tests. *JAMA* **247** (18), 2543–2546 (1982). 7069920

[21] Hosmer, D. W., Lemeshow, S. & Sturdivant, R. X. *Applied Logistic Regression* (Wiley, 2013).

[22] Vickers, A. J. & Elkin, E. B. Decision curve analysis: a novel method for evaluating prediction models. *Med. Decis. Mak.* **26** (6), 565–574 (2006). 10.1177/0272989X06295361 PMC2577036 17099194

[23] Pencina, M. J., D’Agostino, R. B., D’Agostino, R. B. & Vasan, R. S. Evaluating the added predictive ability of a new marker: from area under the ROC curve to reclassification and beyond. *Stat. Med.* **27** (2), 157–172 (2008). discussion 207 – 12. 17569110 10.1002/sim.2929

[24] Shiha, G., Mikhail, N. & Soliman, R. External validation of aMAP risk score in patients with chronic hepatitis C genotype 4 and cirrhosis who achieved SVR following DAAs. *J. Hepatol.* **74** (4), 994–996 (2021). 33340577 10.1016/j.jhep.2020.10.008

[25] Minami, T. et al. Machine learning for individualized prediction of hepatocellular carcinoma development after the eradication of hepatitis C virus with antivirals. *J. Hepatol.* ; (2023). 10.1016/j.jhep.2023.05.042 37716372

[26] Zhang, H., Zhu, J., Xi, L., Xu, C. & Wu, A. Validation of the Toronto hepatocellular carcinoma risk index for patients with cirrhosis in china: a retrospective cohort study. *World J. Surg. Oncol.* **17** (1), 75 (2019). 31039803 10.1186/s12957-019-1619-3 PMC6492382

[27] Åström, H., Ndegwa, N. & Hagström, H. External validation of the Toronto hepatocellular carcinoma risk index in a Swedish population. *JHEP Rep.* **3** (5), 100343 (2021). 34611618 10.1016/j.jhepr.2021.100343 PMC8476346

[28] Shiha, G. et al. International multicenter validation of GES score for HCC risk stratification in chronic hepatitis C patients. *J. Viral Hepat.* **29** (9), 807–816 (2022). 35657138 10.1111/jvh.13717

[29] Abe, K. et al. Multicentre external validation of the GES score for predicting HCC risk in Japanese HCV patients who achieved SVR following DAAs. *Liver Cancer Int.* **2** (3), 102–109 (2021).

[30] Muzica, C. et al. Predictive scores for hepatocellular carcinoma occurrence after hepatitis C virus cure with direct antivirals. *Rev. Med. Chir.* **127** (3), 365–372 (2023).

[31] Soliman, R. et al. Validation of the general evaluation score (GES) for hepatocellular carcinoma risk stratification in chronic hepatitis C genotype 3 patients who achieved SVR in Pakistan [PEER Review ].

[32] Shiha, G. et al. Individualized HCC surveillance using risk stratification scores in advanced fibrosis and cirrhotic HCV patients who achieved SVR: prospective study. *Aliment. Pharmacol. Ther.* **61** (1), 99–108 (2025). 39313490 10.1111/apt.18291

[33] Chang, T. S. et al. Alpha-Fetoprotein measurement benefits hepatocellular carcinoma surveillance in patients with cirrhosis. *Am. J. Gastroenterol.* **110** (6), 836–844 (2015). quiz 845. 25869392 10.1038/ajg.2015.100

[34] Biselli, M. et al. A new approach to the use of α-fetoprotein as surveillance test for hepatocellular carcinoma in patients with cirrhosis. *Br. J. Cancer*. **112** (1), 69–76 (2015). 25314061 10.1038/bjc.2014.536 PMC4453600

[35] Fan, R. et al. Novel, high accuracy models for hepatocellular carcinoma prediction based on longitudinal data and cell-free DNA signatures. *J. Hepatol.* **79** (4), 933–944 (2023). 37302583 10.1016/j.jhep.2023.05.039

