# Supplementary material for: HCC risk stratification scores: insights from large multi-center national cohort study
Source: Sci Rep. 2026 Feb 3;16:4817. doi: 10.1038/s41598-025-34535-w (PMC12873163; doi:10.1038/s41598-025-34535-w)
Supplement: Supplementary file 1 — Supplementary Material 1 [file 41598_2025_34535_MOESM1_ESM.docx]

**HCC risk stratification scores: insights from large multi-center national cohort study**

Imam Waked ^1,2^ , Gamal Esmat ^3^, Mohamed Abdallah ^4^, Aisha Elsharkawy^3^, Wafaa Elakel ^3^, Islam Ammar ^5^, Ehab Kamal ^6^, Mohamed Hassany ^2,7^, Nabiel Mikhail ^8,9^ , Riham Soliman ^9,10^, Wahid Doss ^2,3^ ,Gamal Shiha ^9,11^

*1 National Liver Institute, Menoufia University, Shebeen El Kom, Shebeen El Kom, Egypt*

*2 National* *Committee for Control of Viral Hepatitis, MOH, Egypt*

*3 Endemic Medicine Department, Faculty of Medicine, Cairo University, Cairo, Egypt*

*4 Medical Research Division, National Research Center, Giza, Egypt*

*5 Hepatogastroenterology and Infectious Diseases, Faculty of Medicine, Al-Zhar University*

*6 Medical Research Division, National Research Center, Giza, Egypt*

*7 National Hepatology and Tropical Medicine Research Institute, Cairo, Egypt*

*8 Biostatistics and Cancer Epidemiology Department, South Egypt Cancer Institute,*

*Assiut University, Assiut, Egypt*

*9 Egyptian Liver Research Institute and Hospital (ELRIAH), Mansoura, Egypt*

*10 Tropical Medicine Department, Faculty of Medicine, Port Said University, Port Said, Egypt*

*11 Hepatology and Gastroenterology Unit, Internal Medicine Department, Faculty of Medicine, Mansoura University, Mansoura, Egypt*

**Corresponding authors:**

Prof. Gamal Shiha, Internal Medicine Department, Faculty of Medicine, Mansoura University, Egypt; CEO, Egyptian Liver Research Institute and Hospital (ELRIAH), Mansoura, Egypt.

Email: [g_shiha@hotmail.com](mailto:g_shiha@hotmail.com)

**Supplementary Table (1)**

**Calculation of THRI score** (Sharma et al., 2017) [10]

Point scores are assigned to each covariate (age, sex, etiology and platelets) according to the following table, and the total score is calculated.


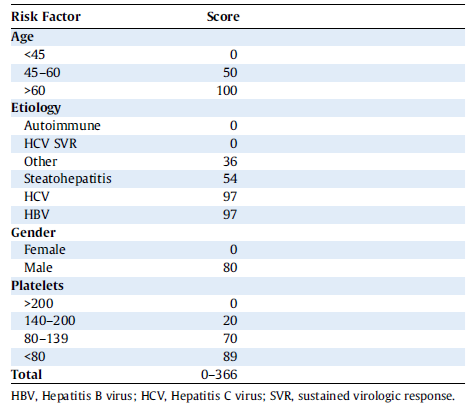


Patients were then stratified into three equally spaced groups based on the THRI score (<120, 120–240, and >240 points).

**Calculation of aMAP score** (Fan et al., 2020) [8]

aMAP risk score = ({0:06 × age + 0:89 × sex (Male: 1; Female: 0)

+0.48 × [(log_10_ bilirubin × 0:66)

+ (albumin × − 0.085)] – 0.01

× platelet} + 7.4 / 14.77 × 100

where age is in year, bilirubin in µmol/l, albumin in g/l and platelets in 103/mm3.

Cut-off values of 50 and 60 were used to separate the cohort into low-, medium and high-risk groups for classification of HCC risk.

**Calculation of GES score** (Shiha et al., 2020) [9]

Point scores are assigned to each covariate according to the following table, and the total score is calculated. Patients are classified as having a low GES risk (≤6 points), an intermediate-risk (>6–7.5 points), or a high-risk score (>7.5 points).

| **Variable** | **score** |
| --- | --- |
| **Sex**  Female  Male | 0  3.5 |
| **Age**  ≤54 years  >54 years | 0  1 |
| **Fibrosis stage**  F0-2  F3  F4 | 0  1.5  3 |
| **Albumin**  ≥ 3.8g/dL  <3.8 g/dL | 0  2 |
| **Alphafetoprotein**  ≤20 ng/ml  >20 ng/ml | 0  3 |
| **Total** | 0–12.5 |

**Calculation of FIB-4 score** (Sterling et al. 2005) [13]

**
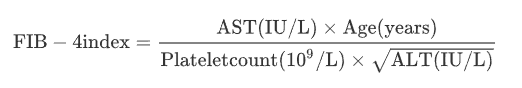
**

**Calculation of ALBI score** (Johnson et al. 2015) [7]

ALBI = (log10 bilirubin × 0.66) + (albumin × -0.085), where bilirubin is in μmol/L and albumin in g/L.

**
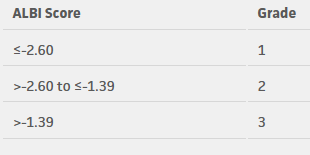
**

**Supplementary Table (2)** Baseline Demographic and Clinical Characteristics of the NCCVH Cohort

| Variable | NCCVH Cohort |
| --- | --- |
| Patient number | 8419 |
| Age (years) | 60.0 (53.0–67.0) |
| Sex   - Males - Females | 3672 (43.6)  4747 (56.4) |
| ALT (U/L) | 42.0 (23.0–65.0) |
| AST (U/L) | 45.0 (30.0–65.0) |
| Total Bilirubin (mg/dL) | 0.90 (0.70–1.30) |
| Albumin (g/dL) | 3.9 (3.5–4.2) |
| Platelets count (/cmm^3^) | 150.0 (122.0–182.0) |
| AFP (ng/ml) | 3.46 (2.30–5.40) |
| Comorbidities   - Yes - No | 1414 (16.8%)  7005 (83.2%) |
| FIB4 | 2.86 (1.82–4.02) |
| Follow up duration (months, mean ± SD) | 25.89 ± 12.89 |
|  |  |

**Supplementary Figure (1)**


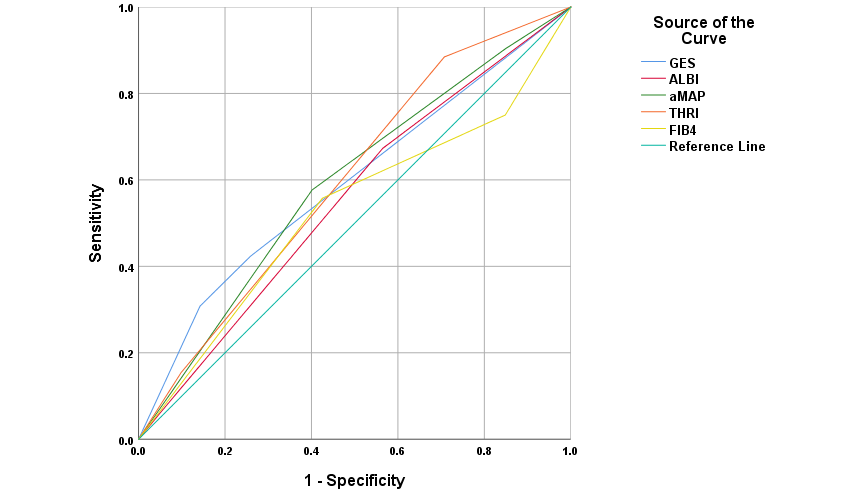


**Supplementary Fig 2**: Receiver Operating Characteristic (ROC) Curves for Risk Scores in Predicting Hepatocellular Carcinoma
